# Muscle strength, functional endurance, and health-related quality of life in active older female golfers

**DOI:** 10.1007/s40520-017-0842-4

**Published:** 2017-10-23

**Authors:** Charlotte Buckley, Maria Stokes, Dinesh Samuel

**Affiliations:** 1 Faculty of Health Sciences, Building 45, University of Southampton, Highfield Campus, Highfield, Southampton, SO17 1BJ UK; 2 Arthritis Research UK Centre for Sport, Exercise and Osteoarthritis, Nottingham, UK

**Keywords:** Active ageing, Muscle strength, Handgrip, Quadriceps, Endurance

## Abstract

**Background:**

Grip strength is a reliable predictor of whole body strength in older adults, but muscle characteristics of people with different activity levels have not been studied previously. The present study examined the relationship between grip strength (GS), quadriceps peak torque (QPT), functional endurance, and health-related quality of life (HRQoL) in older female golfers.

**Methods:**

Twenty-nine healthy female golfers (mean age 69.1 years, SD 3.4) participated. The ISOCOM and JAMAR dynamometers were used to assess QPT and GS, respectively. Functional endurance tests included 1-min sit-to-stand test (1MSTS), 30-s wall press (30SWP), and 2-min stair climb (2MSC). HRQol was assessed using the SF-36 questionnaire.

**Results:**

Mean GS and QPT were 27.5 ± 4 kg/f and 103.7 ± 25.1 N m, respectively. Mean scores for the 1MSTS, 30SWP, and 2MSC were 31 ± 7.7, 17.4 ± 3.5, and 237.5 ± 48.6 repetitions, respectively. GS was moderately correlated with QPT (*r* = 0.44), 1MSTS (*r* = 0.36), and 2MSC (*r* = 0.36), but had weak correlation with 30SWP (*r* = 0.003). Moderate correlation was observed between quadriceps peak torque and the 1MSTS (*r* = 0.50; *p* = 0.01), 2MSC (*r* = 0.44; *p* = 0.02) and 30SWP (*r* = 0.33). 30SWP and 2MSC had moderate correlations with PF *r* = 0.41 (*p* = 0.03) and *r* = 0.61 (*p* < 0.0005) and general physical well-being *r* = 0.47 (*p* = 0.01) and *r* = 0.39 (*p* = 0.04), respectively.

**Conclusion:**

Quadriceps strength was more closely associated with functional endurance than grip strength. A single strength measure may not reflect overall muscle characteristics in active older females, and hence, assessment of both upper and lower limb strengths may be appropriate.

## Introduction

The population is ageing and the proportion of people aged over 65 years is projected to increase from 16% of the total population to 24% by 2051 [1]. Skeletal muscle mass depletes with age in both men and women [2]. By the age of 60, a typical person would have already lost 25% of their youth peak strength [3]. Disuse may be an important cause of muscle atrophy and weakness, rather than ageing alone [4–6], and functional loss may be attenuated by frequent physical activity [7, 8]. The promotion of regular exercise is important for encouraging active ageing to maintain general health. This calls for effective ways to measure muscle strength and endurance in active older individuals. There is also a need to characterise musculoskeletal function in active older female golfers using non-invasive measures, which are easily conducted and reproducible.

Strength is defined as the ability to produce maximal muscle force during a contraction [9, 10]. Lower limb strength is associated with walking performance, ability to get in and out of a chair, step-climbing speed, and rate of falls in older individuals [11]. Strength is also a predictor of various health outcomes such as mortality, future disability, and post-operative complications [12]. A decline in handgrip strength has been associated with an increased risk of morbidity, disability, and mortality, as well as increased healthcare costs [13, 14]. Previous research has suggested that handgrip strength may be a more useful marker of frailty than chronological age [15].

Handgrip and isometric knee extensor strength have been shown to be well correlated and grip strength is often used as a measure of whole body strength in sedentary older adults [16]. Samuel et al. [17] found that expressing upper and lower limb strength as a ratio was a sensitive measure of distinguishing the relative differences between younger and older healthy individuals. The decline in quadriceps strength was found to be greater than the decline in handgrip strength with ageing. It is unknown whether these findings would be seen in active older females and consequently whether handgrip strength would be correlated with quadriceps strength, functional endurance, and HRQoL in this group.

Regular golfing in active older females may have protective effects on muscle function and physical capability. Tsang and Hui-Chan [18] found that male golfers (66.2 ± 6.8 years) had significantly longer duration of single leg stance, less anterioposterior body sway in perturbed single leg stance and lunged further compared to controls (71.3 ± 6.6 years). This suggests that golfers have better static and dynamic balance than non-golfers. Golf is also a weight-bearing exercise which has been shown to prevent age-related decreases in bone mineral density, decreasing the impact of osteoporosis, consequently reducing fracture risk in women of post-menopausal age [19, 20].

Factors other than muscle need to be considered in functional performance. For example, Venturelli et al. [21] reported a significant loss of maximum voluntary contraction in the lower limb of immobile older adults compared to those who were mobile, but in vitro examination of muscle biopsies indicated that intrinsic strength capacity did not differ. These findings suggest that factors other than skeletal muscle, such as neuromuscular control, may cause the loss of function. Unhjem et al. [22] examined the neuromuscular aspects of muscle function and reported that age impacted on efferent drive to the contracting muscle. The decline in efferent drive was mitigated by long-term strength training in master athletes.

The impact of playing golf on muscle strength and endurance has not yet been examined in an active older female population aged 65–80 years. Therefore, the present study aimed to examine the feasibility of investigating the relationship between grip strength and quadriceps strength, functional endurance, and health-related quality of life (HRQoL) in active older female golfers aged 65–80 years.

## Method

### Participants

Twenty-nine healthy, active females aged 65–80 years were recruited from local golf clubs around the Southampton area. A power calculation confirmed that a sample of 36 participants was required, but due to time constraints and drop-outs, this number could not be recruited. However, intra-class correlation coefficients of 0.97 for the isokinetic dynamometer suggest that a smaller sample size was not detrimental [23] to the rigour of the study. Participants were recruited using poster advertisements and information leaflets. Participants who played golf recreationally one or more times a week (playing > 4 h/week) were then telephone screened using a selected set of questions from the Physical Activity Scale for the Elderly (PASE) to ensure moderate activity levels. Participants were excluded if they had any cognitive impairment, known neurological (Parkinson’s disease, stroke or multiple sclerosis) or musculoskeletal condition (active rheumatoid arthritis) or severe injury (e.g., fracture) incurred to the upper, lower limbs or spine in the previous 5 years. Those under current medical treatment affecting muscles, such as long-term steroid use, psychotrophics or muscle relaxants, were also excluded. Those with uncontrolled pain from osteoarthritis, diabetes mellitus, or high blood pressure were excluded from the study, but participants with these conditions controlled were included, to ensure that the sample was representative of a healthy active older population. A summary of participant characteristics is presented in Table 1. Ethical approval was obtained from the Faculty of Health Sciences Ethics Committee at the University of Southampton. All participants provided written informed consent.

**Table 1:** Participant characteristics

	Mean (± SD) (*n* = 29)	Range
Age (years)	69.1 (3.4)	65–77
Height (m)	1.6 (0.1)	1.52–1.73
Weight (kg)	64 (5.4)	50–72
BMI (kg/cm^2^)	24.8 (2.5)	19.4–29.7

### Testing protocol

Prior to testing, participants completed an SF-36 questionnaire used to measure health-related quality of life (HRQoL). The order of testing was not randomised and participants underwent testing of their dominant upper and lower limbs. Limb dominance was assessed by asking participants which hand they would predominantly write or the leg used to kick a football. Height and weight were measured using a stadiometer and scales. All equipment was calibrated by the manufacturers and checked prior to, during, and after study completion.

All participants completed a submaximal warm up prior to strength and endurance measurements. The test procedure was standardised and conducted in the following order: quadriceps peak torque, grip strength, and functional endurance, to ensure that fatigue was not a confounding variable when testing strength. Data collection was carried out by the same investigator (CB) in the Faculty of Health Sciences, University of Southampton.

### Quadriceps strength

**Fig. 1:**
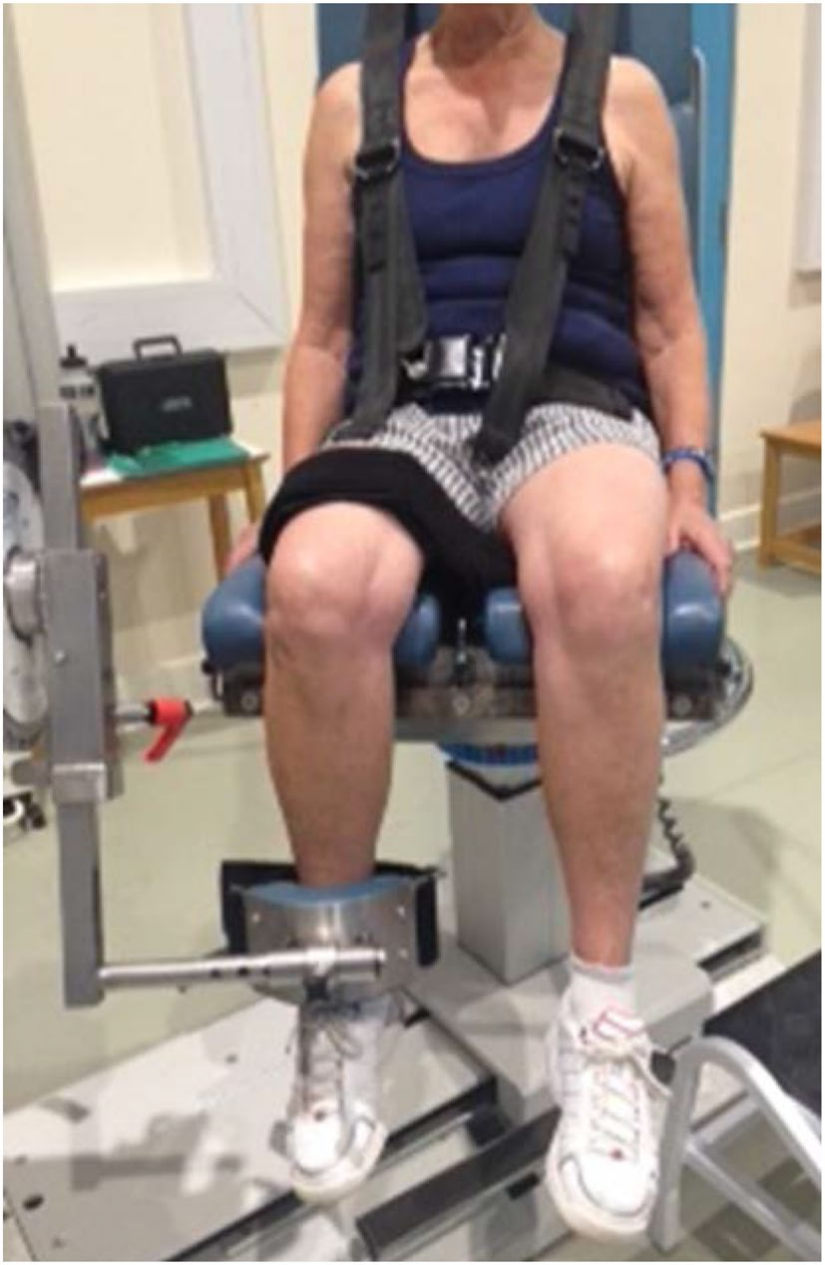
Knee extensor strength testing using isokinetic dynamometer

The ISOCOM isokinetic dynamometer (Eurokinetics Limited, UK) was used to assess quadriceps strength, specifically isometric peak torque. The ISOCOM isokinetic dynamometer uses a conventional rotary torque transducer that is mounted to the rotating axis of the dynamometer. The signal from the transducer is recorded via an A/D card and converted to a torque value automatically. As the transducer is in line with the axis of rotation and the point of resistance on the anterior lower leg (just proximal to the lateral malleolus) was standardised in all participants, it is not necessary to measure lower leg (lever arm) length to determine torque. A calibration check was performed as required by the manufacturer’s specification. Participants were seated, with their upper body strapped firmly against the back rest of the chair, with their hips and knees at 90° and straps tightened across their chest, hips, and thighs, as shown in Fig. 1. Quadriceps strength testing consisted of three maximal isometric knee extensions of the dominant leg. Contractions were held for 5 s with a 30 s rest between repetitions. A count down system on the computer screen was used to ensure that participants were contracting their quadriceps muscle at the correct time, as well as standardised verbal commands from the researcher. The maximum peak torque was used for analysis. Intra-rater reliability testing of the procedure was conducted comparing results collected on two different days from seven volunteers. An intra-class correlation coefficient (ICC model_1,1_) of 0.97 was noted for quadriceps peak torque. A Bland–Altman plot showed a very good level of agreement between sessions for quadriceps peak torque (mean difference: − 5.49; 95% CI 0.86, 0.99).

### Grip strength

Grip strength was measured using a Jamar dynamometer. The test–retest reliability of this measure has been found to be high in older adults (ICC ≥ 0.85) [24]. Participants were seated with their shoulders at 0° abduction and neutral rotation, elbows at 90° flexion and their forearms in a neutral position, as recommended by the American Society of Hand Therapy [25]. The second handle position was used and participants completed three maximal repetitions of grip strength using their dominant and non-dominant hand. Maximal isometric contractions of grip strength were held for 5 s with a 30-s rest between repetitions. Standardised verbal encouragement was used to motivate participants. The maximum value was used in the analysis.

### Functional endurance

Functional endurance was examined using three functional tests, consisting of: 1-min sit-to-stand test (1MSTS) [26], 30-s wall press (30SWP) [27], and 2-min stair climb (2MSC) [28]. For the 1MSTS, a chair was placed firmly against a wall. Participants were asked to sit on the chair with their back straight, arms crossed over the chest, and feet flat on the floor. On the command ‘go’, the participants rose from sitting to standing as many times as possible. If, at 1 min, the participant was half way up, this was counted as a full stand. For the 30SWP, the participant was asked to stand facing the wall with their arms outstretched in front of them and their hands flat on the wall and wrists at 90° of extension, and their feet were arm’s length from the wall, hip width apart. The participant was then asked to complete as many wall presses as possible in 30 s. A complete wall press was when the participant achieves 170° elbow flexion and the forearms became flush with the wall. If, at 30 s, the participant was half way through a wall press, this was counted as a complete wall press. The 2MSC test was conducted at their own pace. The participant was instructed to climb two flights of six steps as many times as possible in 2 min, this involved going up and down the stairs repeatedly. Participants were advised to hold onto a rail on their left for safety reasons. Standardised verbal encouragement was used for all tests.

### Data analysis

Data were analysed using SPSS version 19.0. Normal distribution was assessed using histograms and data were found to be normally distributed. Means, standard deviations, and ranges were used for descriptive statistics, and Pearson’s correlation coefficient was used to examine the relationship between grip strength, quadriceps strength, and the functional endurance tests. Non-parametric data were analysed using Spearman’s correlation. This examined the relationship between the SF-36 scores and quadriceps strength, grip strength, and functional endurance measures. Correlations were categorised as strong (*r* ± 0.70–1.00), moderate (*r* ± 0.30–0.69), or weak (*r* ± 0.00–0.29) [29]. Statistical significance was accepted at *p* = < 0.05. Linear regression was used to assess the magnitude of accountability maximum dominant handgrip strength had for quadriceps strength and functional endurance measures.

## Results

A summary of grip and quadriceps strength and functional endurance tests can be seen in Table 2. Pearson’s correlation presented in Table 3 showed moderate association between quadriceps peak torque and grip strength in active older females (*r* = 0.44; *p* = 0.02).

**Table 2:** Summary of muscle strength and endurance

	Mean (± SD)	Range
Grip strength (kg/f)	27.5 (4)	20–36
Quadriceps torque (N m)	103.7 (25.1)	54.1–140.1
1MSTS	31 (7.7)	17–54
30SWP	17.4 (3.5)	12–26
2MSC	237.5 (48.6)	126–336

**Table 3:** Pearson’s correlation coefficients (*r* values) for knee extensor peak torque, grip strength, and functional endurance tests

	Grip strength	1MSTS	30SWP	2MSC
Quadriceps torque	0.44 (0.02*)	0.50 (0.01*)	0.33 (ns)	0.44 (0.2*)
Grip strength		0.36 (ns)	0.00 (ns)	0.36 (ns)

*ns* not significant

*Correlation at *p* < 0.05

Lower limb endurance tests were significantly correlated with quadriceps peak torque. Moderate correlation was observed between quadriceps peak torque and the 1MSTS (*r* = 0.50; *p* = 0.01), 2MSC (*r* = 0.44; *p* = 0.02), and 30SWP (*r* = 0.33). Grip strength showed moderate correlation with the 1MSTS (*r* = 0.36) and 2MSC (*r* = 0.36), and had the weakest correlation with the upper limb endurance measure, 30SWP (*r* = 0.003). There was a strong correlation between the upper and lower limb endurance measures themselves. A strong correlation was observed between 1MSTS and 30SWP (*r* = 0.70; *p* = 0.0005). 1MSTS showed moderate correlation with 2MSC (*r* = 0.57; *p* = 0.0005).

Age was negatively correlated with grip strength, quadriceps peak torque, and the endurance measures. Quadriceps peak torque was found to be more closely correlated to age than grip strength, with a correlation coefficient of − 0.51 (*p* = 0.004) and − 0.34, respectively (*p* = 0.07). Age showed moderate correlation with 1MSTS (*r* = − 0.31) and 2MSC (*r* = − 0.33) and weak correlation with 30SWP (*r* = − 0.18).

Spearman’s correlation presented in Table 4 showed that grip strength had moderate correlation with general physical well-being (PCS) (*r* = 0.39; *p* = 0.04). Both the 30SWP and 2MSC had moderate correlations with PF *r* = 0.41 (*p* = 0.03) and *r* = 0.61 (*p* < 0.0005) and physical component score *r* = 0.47 (*p* = 0.01) and *r* = 0.39 (*p* = 0.04), respectively. There was moderate correlation between 30SWP and BP *r* = 0.42 (*p* = 0.02) and 2MSC and GH *r* = 0.60 (*p* = 0.00).

**Table 4:** Summary of Spearman’s correlation coefficients for SF-36, quadriceps peak torque, grip strength, and functional endurance tests

	Physical function	Role physical	Bodily pain	General health	Vitality	Social function	Role emotional	Mental health	PCS	MCS
Quadriceps peak torque	0.29 (ns)	− 0.12 (ns)	0.23 (ns)	0.29 (ns)	− 0.01 (ns)	0.03 (ns)	− 0.41 (ns)	0.08 (ns)	0.27 (ns)	0.02 (ns)
Grip strength	0.23 (ns)	0.03 (ns)	0.27 (ns)	0.30 (ns)	− 0.08 (ns)	− 0.03 (ns)	− 0.02 (ns)	− 0.12 (ns)	0.39 (0.04*)	− 0.08 (ns)
1MSTS	0.29 (ns)	− 0.04 (ns)	0.28 (ns)	0.30 (ns)	0.17 (ns)	0.20 (ns)	0.20 (ns)	0.01 (ns)	0.31 (ns)	0.08 (ns)
30SWP	0.41 (0.03*)	− 0.03 (ns)	0.42 (0.02*)	0.22 (ns)	0.27 (ns)	0.10 (ns)	0.01 (ns)	− 0.03 (ns)	0.47 (0.01*)	− 0.03 (ns)
2MSC	0.61 (0.00**)	0.07 (ns)	0.24 (ns)	0.60 (0.00**)	0.30 (ns)	0.27 (ns)	0.28 (ns)	0.32 (ns)	0.39 (0.04*)	0.31 (ns)

*ns* not significant

*Correlation significant at *p* < 0.05

Regression analysis was conducted to estimate the influence grip strength had on quadriceps peak torque and the endurance tests. Linear regression between grip strength and quadriceps peak torque provided an *R* ^2^ value of 0.2. Other linear regressions conducted on 1MSTS, 30SWP, and 2MSC indicated a poor relationship with grip strength *R* ^2^ = 0.10, *R* ^2^ = 0.0, and *R* ^2^ = 0.10, respectively.

## Discussion

The present study provides novel data on the relationships between grip strength, quadriceps strength, functional endurance, and HRQoL in active older female golfers between the ages of 65 and 80. The previous literature highlights that grip strength is well correlated with quadriceps strength in healthy adults [16], confirming that grip strength can be used as a surrogate for quadriceps strength. Handgrip measurements have been widely used in clinical practice to establish sarcopenia over other lower limb measurements [28]. The key reason being, measuring grip strength by handgrip dynamometry is easy to administer, accessible, and cost-effective. Grip strength is associated with a number of health parameters, as well as strength, which suggests an interchangeability of upper and lower limb measurements [28].

### Grip strength and quadriceps peak torque

The present study found a moderate correlation between grip strength and quadriceps strength (*r* = 0.44; *p* = 0.02). The mean handgrip and quadriceps strength were in keeping with those reported by Abe et al. [30] involving 21 active Japanese women with a mean age of 74 ± 3 years (mean handgrip strength: 24.1 ± 4.6 kg, Mean quadriceps strength: 94 ± 23 N m). Recent research conducted by Martien et al. [31] found a positive correlation between handgrip and knee extensor strength (*r* = 0.67) in 947 participants with a mean age of 72.6 (± 8.2) years, 68.2% of which were women. They found that knee extensor strength was not able to predict functional outcomes better than handgrip strength in community-dwelling older adults and nursing home residents. In addition, a positive correlation was found between handgrip strength and the 6-min walk distance (6MWD) (*r* = 0.59; *p* < 0.001) and mPPT, a functional test battery including upper extremity performance (*r* = 0.49; *p* < 0.001). This suggests that handgrip is well correlated with functional lower extremity strength and supports the notion that they are a common underlying construct in community-dwelling individuals [16].

### Muscle strength and functional endurance with ageing

Both grip strength and quadriceps peak torque were negatively correlated with age. Samuel et al. [17] found that quadriceps strength was 25% higher than grip strength in younger adults (grip/quadriceps ratio of 0.75) and the strength of these muscle groups became similar with increasing age, with a grip/quadriceps strength ratio of 1:1 observed in the older groups. This demonstrates that declines in quadriceps strength exceed declines in grip strength with increasing age. The current study could not confirm that quadriceps strength declines at a faster rate than grip strength as controls were not included. However, other research [32] recorded isokinetic strength values of the knee and elbow extensors and flexors in older men (*n* = 9) and reported decreased isokinetic strength in the lower limbs compared to the upper limbs. Lynch et al. [33] also found that peak knee muscle torque (combined extensors and flexors) declined more with age than elbow muscles (combined extensors and flexors), although this relative decline was not calculated.

When comparing sedentary individuals to those who participate in a small amount of physical activity regularly, large functional gains can be observed [34]. A systematic review and meta-analysis conducted by Volkers et al. [35] suggested that regular physical activity is necessary for maintaining strength with advancing age. Individuals who participated in habitual physical activity (*n* = 195) showed a decreased decline in knee extensor strength (*d* = − 0.46; 95% CI 1.07–0.14; *p* = 0.13) compared to sedentary individuals (*n* = 146) who had a significant decline in knee extensor strength within 5–11 years (*d* = − 0.35; 95% CI 2.14 to − 0.55; *p* = 0.001).

In the present study, functional endurance measures had an inverse relationship with age. The magnitude of association between the functional endurance measures with age was moderate for the 1MSTS and 2MSC with Pearson’s correlation coefficients of − 0.31 and − 0.36, respectively. Weak association was found between 30SWP and age (*r* = − 0.18). This functional endurance measure utilizes stronger global muscles of the upper limb such as deltoids, triceps, and biceps working both concentrically and eccentrically throughout one repetition. Therefore, these muscles may be less susceptible to a decline in muscle mass with age.

A moderate correlation was found between quadriceps peak torque and the 1MSTS (*r* = 0.50).This suggests that the 1MSTS may be a good functional method of measuring lower limb strength in active older female golfers. The 1MSTS has been shown to be less valid than the 30-s sit-to-stand test which had high test–retest reliability across sessions in a study assessing lower body strength in community residing older women (*r* = 0.92) [36, 37], but may be more appropriate for an active population. Convergent validity of the 1MSTS is supported by correlations between repetitions of the 6-min walk distance and distance covered in patients with chronic obstructive pulmonary disease (*r* = 0.75) and by healthy individuals (*r* = 0.54) [26]. The mean score for the 1MSTS was 15 ± 5 repetitions in the COPD group, 20 ± 4 repetitions in the healthy group compared to 31 ± 7.7 repetitions in female golfers. This highlights the potential benefit of playing golf in retirement, with the golfing group achieving considerably increased mean sit-to-stand scores compared to other groups. The present data may help provide normative values for healthy active older women between the ages of 65–80 years; however, further research is warranted in a larger sample size.

A moderate correlation was found between the 2MSC and quadriceps peak torque (*r* = 0.44) and 1MSTS (*r* = 0.57). A cross-sectional study conducted by Zech et al. [38] concluded that stair climb and sit-to-stand transfer power were sensitive enough to distinguish between non-frailty and pre-frailty in 29 community-dwelling adults over the age of 65 years. Another study explored the relationship between the stair climb power test (SCPT) and leg power impairments in mobility-limited older adults with a mean age of 75.4 ± 6.9 years who were predominantly women (69%) [39]. Stair climb power per kilogram was moderately associated with dual leg press 40% and dual leg press 70% measured by pneumatic isotonic resistance machines. All these leg power measures correlated with the components of the short physical performance battery (SPPB) score (gait speed, chair stand, and standing balance) excluding the dual leg press measures with standing balance. SCPT most strongly predicted the chair stand aspect of the SPPB (*R* ^2^ = 0.25) compared to dual leg press 40% (*R* ^2^ = 0.12) and 70% (*R* ^2^ = 0.13). Overall, these findings are in line with our own, and, although not directly comparable, suggest that both the 1MSTS and the 2MSC are effective in testing for lower limb strength and endurance in an active older female population.

Grip strength showed moderate correlation with 1MSTS and 2MSC, but had weak correlation with the 30SWP, the upper limb endurance measure. Stevens et al. [40] found that a kilogram increase in grip strength in older community-dwelling women (mean age 68.1 ± 2.5) was associated with improvements in lower limb functional ability; however, upper limb functional ability was not examined. A weak association between grip strength and the 30SWP may be due to the different muscles utilized in gripping. The muscles utilized in grip strength (predominantly hand flexors) are very different to those muscles used during a wall press (deltoid, biceps and triceps) and thus may not have any correlation to each other. Regression analysis revealed that only 10% (*R* ^2^) of the variance in 1MSTS and 2MSC and 4% (*R* ^2^) of the variance in 30SWP could be attributed to grip strength. These results suggest that grip strength could not be used as a measure to predict upper and lower limb functional endurance in this population.

### HRQoL, muscle strength, and functional endurance

In the present study, HRQoL was assessed using the SF-36 questionnaire. Quadriceps peak torque had weak correlations with all SF-36 domain scores and summary scores. Grip strength showed moderate correlations with general health and PCS but had weak correlations with all other domain scores and MCS. This may suggest that grip strength may impact on general health and PCS in active older female golfers, potentially influencing physical function and well-being. However, due to a lack of cause and effect establishment, these findings must be interpreted with caution. Similar results were found by Samuel et al. [41], who studied 84 male and female healthy older adults (mean age 73.2 ± 7.3), and reported a significant correlation with muscle strength and physical function, bodily pain, vitality, social functioning, and role emotional. However, the inability to demonstrate cause and effect highlights the possibility that decreased HRQoL with increasing age may lead to decreased strength rather than the reverse. Alternatively, older adults who had more energy and less bodily pain are likely to participate more in life, fulfilling various societal roles, improving their physical, social, and emotional function. Their study concluded that loss of muscle strength was associated with poorer functional ability, both of these aspects being associated with reduced HRQoL.

The 30SWP showed moderate correlations with physical function, bodily pain, and physical component score. The 2MSC had moderate correlations with physical function, general health, PCS, and MCS. The 1MSTS showed moderate correlation with general health and PCS. This suggests that these easily conducted, functional endurance measures may be useful alongside measures of muscle strength.

## Limitations

The main limitation of this study was the small sample size. More robust results may have been found if a larger sample was utilized. Another limitation was that the units of measurement used in this study for handgrip and quadriceps strength were not the same; therefore, a ratio of handgrip to quadriceps strength could not be calculated. Quadriceps peak torque measured on the dynamometer may not be used in the community, and therefore, this measurement is less reproducible in a community setting. The 6-min walk test is a useful measure of endurance and could be considered for future studies. The order of testing was not randomised. However, in the present study, this was not possible due to the requirements of the other studies being conducted. The lack of data on muscle mass is recognised as a limitation of this study, although muscle strength is known to be highly correlated with muscle size [42]. The absence of a comparative group of sedentary older adults is another important limitation, which should be addressed in future studies. The cross-sectional nature of this study does not enable the cause and effect to be studied but indicates measures that could be used in a prospective study in novice golfers compared with non-golfing controls.

## Conclusions

Functional endurance was more closely related to quadriceps strength than grip strength. It is recommended that when assessing strength and endurance in an active older population a whole body approach to testing may be taken. By assessing quadriceps strength, handgrip strength, and functional endurance, more specific detection of upper and lower extremity strength decline can be achieved. This allows for specific muscle groups to be targeted that may be declining in strength. In the present study, the musculoskeletal function in active older female golfers was characterised. Playing golf after the age of 65 years might be valuable in preserving strength, endurance, and health-related quality of life in active older females.

## References

[1] Lloyd L, Tanner D, Milne A (2014). Look after yourself: active ageing, individual responsibility and the decline of social work with older people in the UK. Eur J Soc Work.

[2] Faulkner JA, Davis CS, Mendias CL (2008). The aging of elite male athletes: age-related changes in performance and skeletal muscle structure and function. Clin J Sport Med.

[3] Frontera WR, Hughes VA, Fielding RA (2000). Aging of skeletal muscle: a 12-yr longitudinal study. J Appl Physiol.

[4] Peterson MD, Rhea MR, Sen A (2010). Resistance exercise for muscular strength in older adults: a meta-analysis. Ageing Res Rev.

[5] Wroblewski AP, Amati F, Smiley MA (2011). Chronic exercise preserves lean muscle mass in masters athletes. Phys Sportsmed.

[6] Wall BT, Dirks ML, van Loon LJ (2013). Skeletal muscle atrophy during short-term disuse: implications for age-related sarcopenia. Ageing Res Rev.

[7] Fiatarone MA, O’Neill EF, Ryan ND (1994). Exercise training and nutritional supplementation for physical frailty in very elderly people. N Engl J Med.

[8] Faulkner JA, Larkin LM, Claflin DR (2007). Age-related changes in the structure and function of skeletal muscles. Clin Exp Pharmacol Physiol.

[9] Rodosky MW, Andriacchi TP, Andersson GB (1989). The influence of chair height on lower limb mechanics during rising. J Orthop Res.

[10] Puthoff ML, Nielsen DH (2007). Relationships among impairments in lower-extremity strength and power, functional limitations, and disability in older adults. Phys Ther.

[11] Fukagawa NK, Wolfson L, Judge J (1995). Strength is a major factor in balance, gait, and the occurrence of falls. J Gerontol A Biol Sci Med Sci.

[12] Bohannon RW (2008). Hand-grip dynamometry predicts future outcomes in aging adults. J Geriatr Phys Ther.

[13] Janssen I, Shepard DS, Katzmarzyk PT (2004). The healthcare costs of sarcopenia in the United States. J Am Geriatr Soc.

[14] Rantanen T, Harris T, Leveille SG (2000). Muscle strength and body mass index as long-term predictors of mortality in initially healthy men. J Gerontol A Biol Sci Med Sci.

[15] Syddall H, Cooper C, Martin F (2003). Is grip strength a useful single marker of frailty?. Age Ageing.

[16] Bohannon RW, Magasi SR, Bubela DJ (2012). Grip and knee extension muscle strength reflect a common construct among adults. Muscle Nerve.

[17] Samuel D, Wilson K, Martin HJ (2012). Age-associated changes in hand grip and quadriceps muscle strength ratios in healthy adults. Aging Clin Exp Res.

[18] Tsang WWN, Hui-Chan CWY (2010). Static and dynamic balance control in older golfers. J Aging Phys Act.

[19] Kelley GA, Kelley KS, Tran ZV (2002). Exercise and lumbar spine bone mineral density in postmenopausal women a meta-analysis of individual patient data. J Gerontol A Biol Sci Med Sci.

[20] Polidoulis I, Beyene J, Cheung AM (2012). The effect of exercise on pQCT parameters of bone structure and strength in postmenopausal women—a systematic review and meta-analysis of randomized controlled trials. Osteoporos Int.

[21] Venturelli M, Saggin P, Muti E (2015). In vivo and in vitro evidence that intrinsic upper- and lower-limb skeletal muscle function is unaffected by ageing and disuse in oldest-old humans. Acta Physiol (Oxf).

[22] Unhjem R, Nygard M, van der Hoven LT (2016). Lifelong strength training mitigates the age-related decline in efferent drive. J Appl Physiol.

[23] Bonett DG (2002). Sample size requirements for testing and estimating coefficient alpha. J Educ Behav Stat.

[24] Wang C-Y, Chen L-Y (2010). Grip strength in older adults: test-retest reliability and cutoff for subjective weakness of using the hands in heavy tasks. Arch Phys Med Rehabil.

[25] Mathiowetz V, Rennells C, Donahoe L (1985). Effect of elbow position on grip and key pinch strength. J Hand Surg.

[26] Ozalevli S, Ozden A, Itil O (2007). Comparison of the sit-to-stand test with 6 min walk test in patients with chronic obstructive pulmonary disease. Respir Med.

[27] Lubans DR, Smith JJ, Harries SK (2014). Development, test–retest reliability, and construct validity of the resistance training skills battery. J Strength Cond Res.

[28] Cruz-Jentoft AJ, Baeyens JP, Bauer JM (2010). Sarcopenia: European consensus on definition and diagnosis Report of the European Working Group on Sarcopenia in Older People. Age Ageing.

[29] Jackson S (2010). Research methods: a modular approach, (Chap. 3).

[30] Abe T, Thiebaud RS, Loenneke JP (2014). Association between forearm muscle thickness and age-related loss of skeletal muscle mass, handgrip and knee extension strength and walking performance in old men and women: a pilot study. Ultrasound Med Biol.

[31] Martien S, Delecluse C, Boen F (2014). Is knee extension strength a better predictor of functional performance than handgrip strength among older adults in three different settings?. Arch Gerontol Geriatr.

[32] Candow DG, Chilibeck PD (2005). Differences in size, strength, and power of upper and lower body muscle groups in young and older men. J Gerontol A Biol Sci Med Sci.

[33] Lynch N, Metter E, Lindle R (1999). Muscle quality. I. Age-associated differences between arm and leg muscle groups. J Appl Physiol.

[34] Concannon LG, Grierson MJ, Harrast MA (2012). Exercise in the older adult: from the sedentary elderly to the masters athlete. PM&R.

[35] Volkers KM, de Kieviet JF, Wittingen HP (2012). Lower limb muscle strength (LLMS): why sedentary life should never start? A review. Arch Gerontol Geriatr.

[36] Jones CJ, Rikli RE, Beam WC (1999). A 30-s chair-stand test as a measure of lower body strength in community-residing older adults. Res Q Exerc Sport.

[37] Rikli RE, Jones CJ (1999). Functional fitness normative scores for community-residing older adults, ages 60–94. J Aging Phys Act.

[38] Zech A, Steib S, Freiberger E (2011). Functional muscle power testing in young, middle-aged, and community-dwelling nonfrail and prefrail older adults. Arch Phys Med Rehabil.

[39] Bean JF, Kiely DK, LaRose S (2007). Is stair climb power a clinically relevant measure of leg power impairments in at-risk older adults?. Arch Phys Med Rehabil.

[40] Stevens P, Syddall H, Patel H (2012). Is grip strength a good marker of physical performance among community-dwelling older people?. J Nutr Health Aging.

[41] Samuel D, Rowe P, Hood V (2012). The relationships between muscle strength, biomechanical functional moments and health-related quality of life in non-elite older adults. Age Ageing.

[42] Moore AZ, Caturegli G, Metter EJ (2014). Difference in muscle quality over the adult life span and biological correlates in the Baltimore longitudinal study. of Aging JAGS.

